# O12.4. SOME OF THE INDIVIDUAL DIFFERENCES IN RISK TO DEVELOP PSYCHOSIS AMONG CANNABIS USERS CAN BE EXPLAINED BY WHERE THEY LIVE AND BY THEIR AGE AT FIRST USE

**DOI:** 10.1093/schbul/sby015.272

**Published:** 2018-04-01

**Authors:** Marta Di Forti, Diego Quattrone, Giada Tripoli, Caterina La Cascia, Laura Ferraro, Charlotte Gayer-Anderson, Craig Morgan, Robin Murray

**Affiliations:** 1 Institute of Psychiatry, Psychology & Neuroscience, King’s College London; 2 University of Palermo

## Abstract

**Background:**

Cannabis use remains the most widely used recreational drug worldwide. Following from several USA states legalisation policies, European countries are reconsidering their cannabis laws. While a significant amount of Epidemiological evidence has reported that cannabis use increases the risk of psychosis it is still unclear: 1) what underpins individual differences in developing a psychotic disorder following cannabis use; 2) if variations in availability of cannabis have affected rate of Psychotic disorders across Europe.

**Methods:**

Using detailed data on lifetime pattern of cannabis use from the EUGEI first episode case-control study (N=2300) and the available Incidence rates of Psychosis calculated for each European site of the same study, we aim 1) to estimate if differences in age at first use, especially of high potency cannabis among cannabis users resulted in differences in their probability to develop psychosis across the study sites; 2) to calculate the proportion of new cases of psychosis attributable to early adolescence-high Potency cannabis in the 5 countries; 3) to relate data on prevalence of cannabis use in each study site with the corresponding Incidence rates for psychotic disorders.

**Results:**

Cannabis users starting using cannabis at age 15 and younger who live in those EU countries where high potency cannabis is available have the highest probability to develop psychosis, compared to never users (Adj ORs from 2.6–5.9; p<0.01). Moreover, the proportion of new cases of Psychosis attributable to heavy use started in adolescence was between 20% and 37%.

Finally, the correlation between lifetime use of cannabis in population controls from the study sites was significantly correlated with the corresponding incidence rates for Psychosis (r=0.6; p<0.001)

**Discussion:**

Before Europe rushes into the USA legalisation “moda” more public education effort might need to be invested in reducing the use of high potency type of cannabis among young adolescents. The latter could lead to a significant reduction in the proportion of new cases of psychosis across Europe.

